# Mobility, independent agency, and cosmopolitan settlement: Evidence from Chinese senior undergraduates

**DOI:** 10.3389/fpsyg.2022.1057974

**Published:** 2022-12-16

**Authors:** Hui Tang, Gengyao Chen, Zhijun Liu, Ran Zhao, Cheng Lu, Yanhua Su

**Affiliations:** ^1^School of Education, Zhaoqing University, Zhaoqing, China; ^2^Liri Elementary School, Shantou, China; ^3^Center for Mental Health Research in School of Management, Zunyi Medical University, Zunyi, China; ^4^Harbin Institute of Technology, Shenzhen, China; ^5^Nanfang College Guangzhou, Guangzhou, China

**Keywords:** Chinese, cosmopolitanism, voluntary settlement, independence, relational mobility, residential mobility

## Abstract

Cosmopolitan cities share similarities with historical frontiers, including potential opportunities for economic success, high social mobility, weakened traditional conventions, and adventure and novel experiences. Individuals with high independence typically prefer to settle in cosmopolitan cities. However, previous research testing this cosmopolitan settlement hypothesis did not consider the influence of relational mobility and residential mobility. Moreover, the mechanisms that drive people to prefer cosmopolitan cities remain unclear. This study examines the relationships among independence, relational mobility, residential mobility, and preference for cosmopolitan cities among 296 Chinese senior undergraduates. The results indicate that: (1) independence remains a positive predictor of the preference for cosmopolitan cities above and beyond relational mobility, residential mobility (i.e., history, state, and intention), and other covariates; (2) intention of residential mobility also positively predicts preference for cosmopolitan cities when controlling for related covariates; and (3) relational mobility indirectly predicts perceived preference for cosmopolitan cities through dependence. This research underscores the importance of identifying the factors and mechanisms affecting cosmopolitan settlement.

## 1 Introduction

People are progressively moving to more economically developed regions that provide greater employment opportunities, wages, social resources, self-fulfillment, and novel experiences (Sevincer et al., 2017; Su et al., 2019; Liu and Yuan, 2022). In China, a great number of people are moving from rural regions to metropolitan centers, such as Beijing, Shanghai, Guangzhou, and Shenzhen, as a result of the reform and opening up, as well as urbanization. By the end of 2021, China’s urban population was 914.25 million, with an urbanization rate of 64.72% (Xinhuanet, 2021), and a large number of people are still moving to cities from rural regions every year (Zuo et al., 2022). Based on recent investigations (Zeng et al., 2021; Xie et al., 2022), Chinese college graduates and senior undergraduates tend to choose to work in first- and second-tier cities, or big and medium cities with greater economic levels. In this paper, we consider this development from the perspective of social-ecological psychology and ask why Chinese senior undergraduates exhibit this preference.

### 1.1 Voluntary frontier settlement hypothesis

Studies of the “Western Frontier” in the United States during the 18th and 19th centuries, Japan’s “Northern Frontier” (i.e., Hokkaido), China’s Chuang Guangdong movement and China’s ongoing “Southern Frontier” (i.e., Shenzhen) reveal possible reasons for the preference for cosmopolitan cities among Chinese college graduates and senior undergraduates (Kitayama et al., 2006, 2009; Feng et al., 2017; Bai and Ren, 2021). The voluntary frontier settlement hypothesis not only argues that the frontiers offered more opportunities for individuals to pursue wealth and freedom, attracting a large number of voluntary migrants with independent orientation, but also that these immigrants were an important factor in the formation of an individualistic culture in the chosen settlement regions (Kitayama et al., 2006, 2009). Independent self-construal is an essential component of a perspective of the self that is separate from social context. Individuals with high independence define themselves in accordance with their internal attributes and traits, emphasizing personal qualities and successes (Markus and Kitayama, 1991). Previous studies have shown that people in the western United States, where there were more early pioneers, had a higher independent self-construal than those in the eastern United States, as indicated by a greater emphasis on self-direction, universality, and stimulation (Kitayama et al., 2010); a higher percentage of people living alone; more self-employment (Vandello and Cohen, 1999); and a lower tendency to change their views due to social norms or external pressures (Varnum, 2012). Research has found that the voluntary settlement hypothesis can also be applied to collectivistic Eastern cultures. For instance, in the early 20th century, large numbers of mainland Japanese voluntarily migrated to Hokkaido, Japan’s northernmost island. Hokkaido’s residents have been shown to be more independent than mainland-born Japanese (Kitayama et al., 2006; Ishii, 2014). In China, Chuang Guangdong movement, in which millions of people settled in the northeastern region of China during the 19th and 20th centuries, led to the formation of the China’s “Northeast Frontier.” Research shows that people from the northeast of China (e.g., Heilongjiang) reported more self-centric and less in-group favoritism, and are more likely to give babies unique names than those in Shandong (Bai and Ren, 2021). Moreover, since the 1980s, many Chinese inland residents have willingly moved to Shenzhen, which is situated on the southernmost part of Guangdong Province and is near Hong Kong, seeking better career and economic opportunities. Shenzhen has been considered an ongoing voluntary frontier settlement, and research indicates that Shenzhen’s residents are more independent than those of other mainland cities (e.g., Foshan, Wuhan, and Xiangfan; Chen et al., 2016, 2019; Feng et al., 2017; Luo and Ren, 2018). Overall, these studies provide evidence of the voluntary frontier settlement hypothesis, which holds that individuals who have a strong sense of independence are more willing to relocate to regions with more opportunities to pursue personal wealth and freedom.

### 1.2 Cosmopolitan settlement hypothesis

The western regions of the United States, Hokkaido in Japan, and three provinces in northeast China and Shenzhen in China are all located in the frontier areas of their respective nations, where the physical or social environment is more strenuous than that of one’s hometown. It is becoming increasingly more difficult to find topographical frontiers as a result of rising modernization. Recently, Sevincer et al. (2015) proposed that the cosmopolitan city is the contemporary world’s frontier. Although cosmopolitan cities have larger populations and better infrastructure and institutions than the historical frontiers, the two types of settlements do share some similarities. For instance, they not only provide more opportunities for success, as well as novel and adventurous experiences, but also exhibit high social mobility and few conventional customs (Sevincer et al., 2015). Therefore, researchers have argued that modern cosmopolitan cities are also attractive to individuals with a high independent orientation (Sevincer et al., 2015). Research conducted in Germany (Sevincer et al., 2015) has shown that students who wanted to move to a cosmopolitan city (i.e., Berlin) had higher levels of independent self-construal than those who wanted to move to a smaller city (i.e., Braunschweig). Moreover, priming university students with an independent mindset increased their choice of a cosmopolitan city and their inclination to relocate to a new city. Furthermore, university students who relocated to a cosmopolitan city (i.e., Berlin) had a greater number of personal goals and a higher demand for being unique than their counterparts who relocated to a non-cosmopolitan city (i.e., Braunschweig) and those who stayed put (i.e., local students in Berlin and Braunschweig).

Sevincer et al. (2017, 2021) proposed four distinguishing characteristics of cosmopolitan cities, including social diversity, innovativeness, equality, and more economic opportunities. Using this conceptual framework, they developed the Cosmopolitan City Scale (CCS) to measure how cosmopolitan individuals perceive certain cities (Sevincer et al., 2017). Research based on samples from Western, Educated, Industrialized, Rich, and Democratic (WEIRD) societies has also shown that perceived cosmopolitanism is significantly and positively linked to measures of objective cosmopolitanism (e.g., proportions of self-employed workers, minorities, foreign-born inhabitants, LGBTQ + population, high-tech index, number of museums, and per capita income) and risk-taking (Sevincer et al., 2017, 2021). Moreover, individuals with greater degrees of independent self-construal or risk-taking have been shown to prefer living in cosmopolitan cities. Independent self-construal or risk-taking still predicted preference for cosmopolitan cities after controlling for demographic variables and personality traits (Sevincer et al., 2017, 2021). These findings provided preliminary evidence for the cosmopolitan settlement hypothesis in individualistic Eastern cultures. However, there is scarce evidence supporting the cosmopolitan settlement hypothesis in collectivistic Eastern cultures.

More importantly, researchers have argued that testing the voluntary settlement hypothesis should take mobility into account (Feng et al., 2017; Luo and Ren, 2018). Further, we know little about the association between mobility and preference for cosmopolitan cities. Following this logic, we systematically control for the effects of residential and relational mobility—two socio-ecological characteristics that have received substantial attention (Oishi et al., 2015; Yuki and Schug, 2020; Oishi and Tsang, 2022). Relational mobility refers to the extent to which people can develop new connections and exit from unfavorable relationships (Oishi and Tsang, 2022). In high-relational-mobility societies, individuals have various opportunities to engage with new people, choose which groups to join, and leave long-standing and dissatisfying relationships. In contrast, in low-relational-mobility societies, individuals have less freedom to pick new partners or walk away from current relationships because interpersonal and group connections are more likely to be stable (Schug et al., 2010; Yuki and Schug, 2012; Thomson et al., 2018). Studies have shown that individuals from the high-relational-mobility environment have an increased tendency to be extroverted and to engage in self-improvement (Falk et al., 2009; Kim et al., 2018). It has also been shown that high-relational-mobility promotes the formation and maintenance of voluntary ties with other people, as well as the increased integration of various social networks (Thomson et al., 2018; Li et al., 2022). As cosmopolitan cities are full of social diversity, innovativeness, equality, and economic opportunities, people who live in high-relational-mobility societies are inclined to relocate to cosmopolitan cities.

Residential mobility involves the frequency with which people move from one residence to another (Oishi and Tsang, 2022). As residential mobility often brings about significant changes in the physical and interpersonal environment, it is considered an important socio-ecological factor (Oishi, 2014; Oishi and Tsang, 2022). It has been demonstrated that frequently changing one’s residence affects relationship preferences and patterns, leading a person to favor broader social networks, more open interaction, and lower-commitment groups (Oishi and Tsang, 2022). In turn, relationship styles and preferences might make individuals from high-residential-mobility communities inclined to move to cosmopolitan cities. Research has shown that individuals with high residential mobility tend to have a broader global identity and view themselves as global citizens (Borschel et al., 2019; Wang et al., 2021). Residential mobility also refers to the intention to move in the future (Zuo et al., 2018; Choi and Oishi, 2020). Research has indicated that the expectation of future residential mobility rather than a history of it significantly predicted decreased subjective wellbeing, high insecurity, and materialism among Chinese undergraduates (Meng and Xie, 2019; Zhou and Xie, 2019). Therefore, if we consider the effects of relational and residential mobility, we can expect a robust connection between independent self-construal and a preference for cosmopolitan cities.

Because subjective social status, gender, urban resident, and being an only child may influence one’s residential preferences (Murray, 2012; Wen et al., 2020; Xie et al., 2022), we also controlled for these demographic variables. Moreover, as individuals might choose to relocate to a city that resembles or is near their hometown or the city where their university is located (Sevincer et al., 2015, 2017, 2021), we also measure the objective cosmopolitanism of their hometown and university cities, as well as the distance between their intended destination city and their hometown and university cities. This made it possible to evaluate the association between independent self-construal and preference for a cosmopolitan city above and beyond these covariates.

### 1.3 The current research

In summary, this study first examined the connection between independent self-construal and a preference for cosmopolitan cities among selected Chinese senior undergraduates who are looking for their first job, after factoring in the effects of the abovementioned covariates. We test the following hypothesis:

**Hypothesis 1.** Dependence is linked to an increased preference for cosmopolitan cities above and beyond the effects of demographic variables (i.e., gender, being an only child, urban resident, and subjective social status), the objective cosmopolitanism of one’s hometown and university city, distance between their destination city and both their hometown and university, and relational and residential mobility.

Second, we also investigated the association between mobility (i.e., relational and residential mobility) and a preference for cosmopolitan cities among selected Chinese senior under, after factoring in the effects of the abovementioned covariates and dependence. We test the following hypothesis:

**Hypothesis 2.** Mobility (i.e., relational and residential mobility) is linked to an increased preference for cosmopolitan cities above and beyond the effects of demographic variables (i.e., gender, being an only child, urban resident, and subjective social status), the objective cosmopolitanism of one’s hometown and university city, distance between their destination city and both their hometown and university, and dependence.

Third, the mechanisms that lead to a preference for cosmopolitan cities remain unclear. We propose that independence may account for the links between mobility variables (relational and residential mobility) and preference for cosmopolitan cities. Our hypothesis corroborates prior assertions that mobility might contribute to the formation of one’s independent orientation (Choi and Oishi, 2020; Yuki and Schug, 2020; Oishi and Tsang, 2022). For instance, research has shown that individuals with high residential mobility during their childhood have a tendency to place a greater emphasis on their personal self (e.g., personality traits), while placing less emphasis on their collective self (e.g., group membership; Oishi et al., 2007). Likewise, a recent study also has demonstrated that higher residential mobility can result in greater concern for self-interest, which refers to an instrumental concern with maximizing one’s benefits (Chen et al., 2022). Moreover, high relational mobility has been shown to be positively linked to high independence (Feng et al., 2017; Thomson et al., 2018; San Martin et al., 2019). Therefore, we examined the following hypothesis:

**Hypothesis 3.** Mobility (i.e., relational and residential mobility) have indirect effects on preference for cosmopolitan cities *via* dependence, above and beyond the effects of demographic variables (i.e., gender, being an only child, urban resident, and subjective social status), the objective cosmopolitanism of one’s hometown and university city, and distance between one’s destination city and their hometown and university cities.

## 2 Materials and methods

### 2.1 Participants

Using the convenience sampling strategy, 330 undergraduates from four universities in Guangdong (i.e., Guangzhou and Zhaoqing), Guizhou (i.e., Zunyi), and Heilongjiang (i.e., Harbin) provinces, who were entering their senior year, were invited to complete online questionnaires through Wenjuanxin, an online survey platform, during November and December 2019. Before voluntarily answering a series of survey questions, the participants completed permission forms and were assured that their responses would be confidential and used only for academic purposes. First, participants were asked to choose the top two cities in which they most preferred to live and work after graduation. We also used the CCS to assess each city’s perceived cosmopolitanism. Then, participants completed the Singelis Self-Construal Scale, the Relationship Mobility Scale, the Scale of Personal Residential Mobility, and demographic information (i.e., gender, age, siblings, ethnicity, highest educational attainment of parents, hometown, and the city where their university is located). Finally, we matched the objective cosmopolitanism of participants’ preferred cities, hometowns, and university cities, as well as the distance between their destination city and hometown or university cities. To reduce the impact of random replies or inattention, the online questionnaire contained two validity check questions (e.g., “Please indicate ‘strongly agree’ for this item”). Only individuals who answered these two validity check questions correctly were included in the final sample (*N* = 294, or 89.70%). The participants’ ages varied from 21 to 25 years (M_*age*_ = 21.71 years, SD = 0.77 years), and the majority were female (79.6%, *n* = 234), of Han ethnicity (94.6%, *n* = 278), urban (56.1%, *n* = 165), and had at least one sibling (60.2%, *n* = 177). Regarding their parents’ level of education, the majority had completed secondary education (66.67%, *n* = 196), while the remainder had completed either higher education (22.79%, *n* = 67) or primary education (10.54%, *n* = 31).

### 2.2 Measures

#### 2.2.1 Preference for cosmopolitan cities

The CCS is a unidimensional scale that evaluates an individual’s perceptions of the cosmopolitanism of a city and their preference for cosmopolitan cities (Sevincer et al., 2017). This scale includes nine items, and participants rate each item (e.g., “is tolerant toward minority groups groups”), from 1 (strongly disagree) to 7 (strongly agree). The English version of the CCS was translated into Chinese. In this study, 294 participants used the CCS to evaluate 70 cities in which they most preferred to work. The one-factor model indicated an adequate level of fit to the data (χ^2^ = 386.02, *df* = 27, comparative fit index [CFI] = 0.92, Tucker-Lewis index [TLI] = 0.89, root mean square error [RMSEA] = 0.15). All factor loadings were statistically significant, varying between 0.74 and 0.87. The Cronbach’s alpha coefficient for the top five cities’ (Guangzhou, Shenzhen, Shanghai, Beijing, Hangzhou) CSS total scores ranged from 0.91 to 0.95, and the Cronbach’s alpha coefficient for the CSS total score of all cities was 0.94. In terms of criterion validity, perceived cosmopolitanism was positively correlated with objective cosmopolitanism (i.e., city business attractiveness; *r* = 0.42, *p* < 0.001).

#### 2.2.2 Dependence

The Chinese version of the Singelis Self-Construal Scale was used to explicitly assess independence and interdependence (Singelis, 1994; Feng et al., 2017). This scale includes two subscales of independent (12 items; e.g., “I act the same way no matter who I am with”) and interdependent (12 items; e.g., “It is important for me to maintain harmony within my group”). Each item is evaluated using a seven-point scale, from 1 (strongly disagree) to 7 (strongly agree). In this study, only the independence subscale was used, and higher scores indicate higher independent orientation. The internal consistency coefficient of the independent subscale in this study was 0.71.

#### 2.2.3 Relational mobility

Relational mobility was measured using the 12-item Relationship Mobility Scale developed by Yuki et al. (2007), see also Thomson et al. (2018). This measure asks participants to express their perceptions of other people creating new connections and terminating existing ones in their surrounding society, using a six-point scale from 1 (strongly disagree) to 6 (strongly agree). Example items include, “They have many chances to get to know other people.” Higher scores indicate a higher level of relational mobility in their surrounding society. The internal consistency coefficient of the total scale in this study was 0.87.

#### 2.2.4 Residential mobility

Residential mobility was evaluated using the Scale of Personal Residential Mobility (Zuo et al., 2018). This measure assesses residential mobility from three time perspectives: history (six items; e.g., “I have changed my residence many times from birth to now”), state (six items; e.g., “I have lived in my current residence for a very long time”), and intention (six items; e.g., “Moving to other areas is a good idea”). Participants were asked to rate the statements according to their level of agreement, from 1 (completely disagree) to 7 (completely agree). Cronbach’s alpha values for the history, state, and intention scales were 0.80, 0.73, and 0.80, respectively.

#### 2.2.5 Objective cosmopolitanism of hometown and university cities, and distances between the destination city and the hometown and university cities

We use the ranking of cities’ business attractiveness 2019 in China released by the Rising Lab on May 24, 2019 (The Rising Lab, 2019) to measure objective cosmopolitanism. The rankings consider “the concentration of commercial resources, connectivity, urban residents’ activity, diversity of lifestyle, and future potential based on commercial data from 170 major consumer brands, user behavior data from 17 leading internet firms, and urban big data” (Yicai Global, 2021). Higher business attractiveness indicates a higher level of business development in the city. We matched the business attractiveness of participants’ preferred cities, hometowns, and university cities. We also use the Haversine formula to calculated the distance (*Distance*_*ij*_) between their destination city (City*_*i*_*) and their hometown and university cities (City*_*j*_*) based on each city’s latitude and longitude (Sinnott, 1984; Jensen et al., 2015; Yuan and Zhou, 2021). Each city’s latitude and longitude were collected from Geo Data Source,^1^ and *r* is equals to 3,963 miles.


Distance=i⁢jarccos{cos(latitude)icos(longitude)icos(latitude)jcos(longitude)j+cos(latitude)isin(longitude)icos(latitude)jsin(longitude)j+sin(latitude)isin(latitude)j}2πr/360.

#### 2.2.6 Subjective social status

The MacArthur Scale of Subjective Social Status tests how individuals feel about their own social status by showing them a photograph of a “social ladder” with ten rungs. Individuals are invited to choose the step that most accurately defines their social status in their community. A higher position on the ladder indicates a greater social status. According to previous studies, subjective social status has a positive correlation with one’s income, occupation, and level of education (Operario et al., 2004; Hu et al., 2012).

#### 2.2.7 Analytical strategy

First, we performed descriptive statistics and Pearson correlational analyses between the key variables using SPSS 25.0. Next, to investigate Hypothesis 1 and Hypothesis 2, we performed hierarchical multiple regression analyses using the perceived cosmopolitanism of the first and second favored cities as the dependent variable. Finally, to examine Hypothesis 3, we used Model 4 of the PROCESS macro in SPSS 25.0 (Hayes, 2013). We also performed bootstrapping with 5,000 samples to ascertain the mediation effect. As recommended by researchers (Preacher and Hayes, 2008; Owens and Hekman, 2016), there is a substantial mediation effect at the level of α = 0.05 if the bias-corrected bootstrap 95% confidence interval (CI) does not contain zero.

## 3 Results

### 3.1 Descriptive statistics and pearson correlational analyses

Table 1 provides descriptive statistics of the variables. The perceived cosmopolitanism of the first and second preferred cities was significantly and positively correlated with independence and relationship mobility. The perceived cosmopolitanism of the first preferred city was significantly and negatively associated with history and with state of residential mobility but not intention of residential mobility. The perceived cosmopolitanism of the second preferred city was significantly and negatively linked to state of residential mobility and positively linked to intention of residential mobility. However, the association between the perceived cosmopolitanism of the second preferred city and history of residential mobility was not significant and was close to zero.

**TABLE 1:** Descriptive statistical and correlation analysis for each variable.

Variables	*M*	*SD*	1	2	3	4	5	6	7
1.*CCS*1	5.88	0.88	1						
2.*CCS*2	5.79	0.86	0.52***						
3.*Independence*	4.74	0.67	0.22***	0.21***					
4.*Relationalmobility*	4.85	0.98	0.16**	0.21***	0.35***				
5.*History*	2.60	1.32	−0.13*	–0.07	–0.03	0.06			
6.*State*	2.47	1.06	−0.16**	−0.16**	–0.04	–0.08	0.49***		
7.*Intention*	4.34	1.20	0.10	0.13*	0.05	0.06	0.09	0.08	1

CCS1 = perceived cosmopolitanism of the first preferred city; CCS2 = perceived cosmopolitanism of the second preferred city; History = history of residential mobility; State = state of residential mobility; Intention = intention of residential mobility.

***p < 0.001, **p < 0.01, and *p < 0.05.

### 3.2 Results of regression analyses and mediation analysis

#### 3.2.1 The first preferred city

In the first stage, we entered demographic variables (i.e., gender, being an only child, urban resident, and subjective social status), objective cosmopolitanism of one’s hometown and university cities, and distances between the destination city and the hometown and university cities. These variables explained 8.7% of the variance (Table 2). In the second stage, relational mobility and residential mobility (i.e., history, state, and intention) were added, which explained an additional 6.6% of the variance. As shown in Table 2, state and intention of residential mobility, rather than history of residential mobility and relational mobility, significantly predicted a preference for cosmopolitan cities. In the third stage, independence was added, explaining an additional 2.7% of the variance. As presented in Table 2, independence significantly and positively predicted a preference for cosmopolitan cities. Moreover, when we included dependence in the second stage and relational mobility and residential mobility (i.e., history, state, and intention) in the third stage, dependence explained an additional 4% of the variance, and the mobility variables an additional 5.3%. As described in Table 2, state and intention of residential mobility significantly predicted a preference for cosmopolitan cities above and beyond independence.

**TABLE 2:** Results of hierarchical regression analyses for dependence, mobility, and other covariates predicting preference for cosmopolitan cities (the first preferred city).

Variable	*B*	*SE*	β	*R* ^2^	*CM*	Δ*R*^2^
Step 1				0.087		0.087
Gender (boy = 1, girl = 0)	0.04	0.13	0.02			
Being an only child	0.04	0.12	0.02			
Urban resident	–0.03	0.12	–0.02			
Subjective social status	0.04	0.03	0.08			
CBA of hometown	0.01	0.01	0.25***			
CBA of university	0.01	0.01	0.05			
Distance city-hometown	0.01	0.01	0.21**			
Distance city-university	–0.01	0.01	–0.07			
Step 2a				0.127	2a-1	0.04
Relational mobility	0.10	0.05	0.11			
History of residential mobility	–0.05	0.04	–0.08			
State of residential mobility	–0.12	0.06	−0.15*			
Intention of residential mobility	0.11	0.04	0.15*			
Step 2b				0.153	2b-1	0.066
Independence	0.26	0.07	0.20***			
Step 3				0.305	3-2a	0.053
					3-2b	0.027
Relational mobility	0.04	0.06	0.04			
History of residential mobility	–0.05	0.04	–0.07			
State of residential mobility	–0.13	0.06	−0.15*			
Intention of residential mobility	0.11	0.04	0.15*			
Independence	0.23	0.08	0.18**			

CBA of hometown = city of hometown, business attractiveness; CBA of university = city of university, business attractiveness; Distance city-hometown = distance between the first preferred city and hometown; Distance city-university = distance between the first preferred city and university.

***p < 0.001, **p < 0.01, *p < 0.05.

**FIGURE 1:**
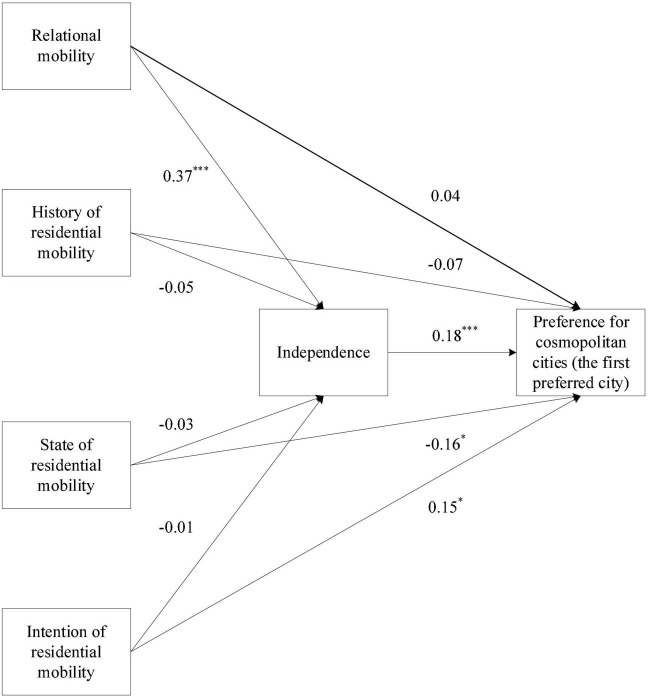
The mediating role of independence in the association between mobility and preference for cosmopolitan cities (the first preferred city), with standardized coefficients. Results of covariates are not shown for the purpose of simplicity; ^***^p < 0.001, *p < 0.05.

The results of Model 4 are presented in Figure 1. Relational mobility (β = 0.37, *p* < 0.001) rather than history (β = −0.05, *p* > 0.05), state (β = −0.03, *p* > 0.05), and intention (β = −0.01, *p* > 0.05) of residential mobility significantly predicted independence. Independence (β = 0.18, *p* < 0.01), as well as state (β = −0.16, *p* < 0.05) and intention (β = 0.15, *p* < 0.05) of residential mobility, rather than history of residential mobility (β = −0.07, *p* > 0.05) and relational mobility (β = 0.04, *p* > 0.05), significantly predicted perceived cosmopolitanism. Furthermore, the results of the mediation analysis demonstrated that the indirect effect of relational mobility on the preference for cosmopolitan cities *via* dependence was statistically significant, as the bias-corrected bootstrap 95% CI excluded zero (β = 0.07; Boot SE = 0.03; 95% CI = [0.01, 0.12]). By contrast, the indirect effect of history (β = −0.01; Boot SE = 0.01; 95% CI = [−0.04, 0.14]), state (β = −0.01; Boot SE = 0.01; 95% CI = [−0.02, 0.03]), and intention (β = 0.01; Boot SE = 0.01; 95% CI = [−0.02, 0.03]) of residential mobility on the preference for cosmopolitan cities through dependence was not statistically significant, as the bias-corrected bootstrap 95% CI included zero.

#### 3.2.2 The second preferred city

Similarly, in the first stage, demographic variables (i.e., gender, being an only child, urban resident, and subjective social status), objective cosmopolitanism of hometown and university cities, and distance between the destination city and the hometown and university cities were included as covariates; they explained 10.9% of the variance. In the second step, the mobility measures were entered and explained an additional 5.7% of the variance. As shown in Table 3, only relational mobility and intention of residential mobility predicted a preference for cosmopolitan cities. In the third stage, independence was added, which explained an additional 1.5% of the variance. As presented in Table 3, independence significantly and positively predicted a preference for cosmopolitan cities. Moreover, when we entered dependence in the second stage and the mobility variables in the third stage, dependence explained an extra 3.1% of the variance, and the mobility variables an additional 4.1%. As described in Table 3, only intention of residential mobility predicted a preference for cosmopolitan cities above and beyond dependence.

**TABLE 3:** Results of hierarchical regression analyses for dependence, mobility, and other covariates predicting preference for cosmopolitan cities (second preferred city).

Variable	*B*	*SE*	β	*R* ^2^	*CM*	Δ*R*^2^
Step 1				0.132		0.132
Gender (boy = 1, girl = 0)	–0.09	0.12	–0.04			
Being an only child	0.32	0.12	0.18**			
Urban resident	–0.24	0.11	−0.14*			
Subjective social status	0.06	0.03	0.13*			
CBA of hometown	0.01	0.02	0.19**			
CBA of university	0.01	0.01	0.02			
Distance city-hometown	0.01	0.01	0.04			
Distance city-university	0.01	0.01	0.22**			
Step 2a				0.190	2a-1	0.057
Relational mobility	0.14	0.05	0.17**			
History of residential mobility	–0.03	0.04	–0.05			
State of residential mobility	–0.08	0.05	–0.10			
Intention of residential mobility	0.09	0.04	0.12*			
Step 2b				0.163	2b-1	0.030
Independence	0.22	0.07	0.18**			
Step 3				0.203	3-2a	0.014
					3-2b	0.041
Relational mobility	0.10	0.05	0.12*			
History of residential mobility	–0.03	0.04	–0.04			
State of residential mobility	–0.08	0.05	–0.10			
Intention of residential mobility	0.09	0.04	0.12*			
Independence	0.16	0.07	0.13*			

CBA of hometown = city of hometown, business attractiveness; CBA of university = city of university, business attractiveness; Distance city-hometown = distance between the second preferred city and hometown; Distance city-university = distance between the second preferred city and university.

**p < 0.01, *p < 0.05.

**FIGURE 2:**
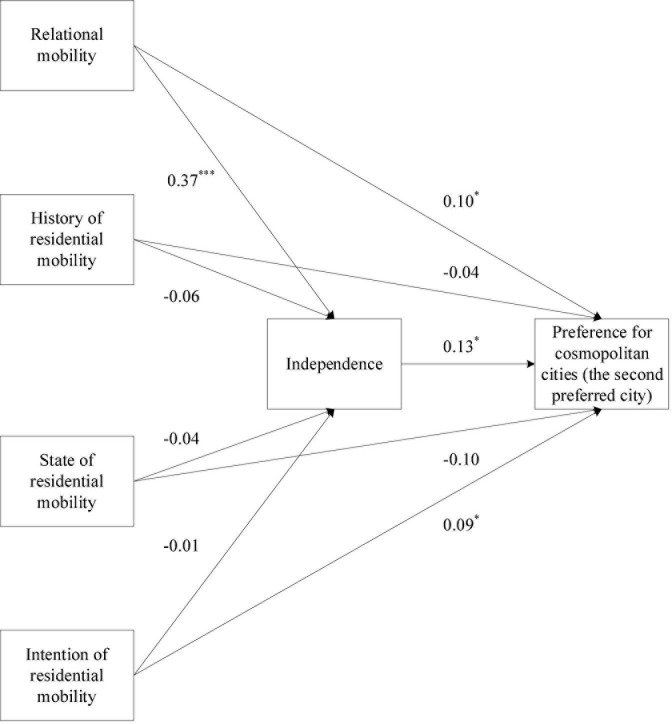
The mediating role of independence in the association between mobility and preference for cosmopolitan cities (the second preferred city), with standardized coefficients. Results of covariates are not shown for the purpose of simplicity; ^***^p < 0.001, *p < 0.05.

The results of Model 4 are shown in Figure 2. Relational mobility (β = 0.37, *p* < 0.001) rather than history (β = −0.06, *p* > 0.05), state (β = −0.04, *p* > 0.05), and intention (β = −0.01, *p* > 0.05) of residential mobility significantly predicted independence. Independence (β = 0.13, *p* < 0.05), relational mobility (β = 0.10, *p* < 0.05), and intention of residential mobility (β = 0.09, *p* < 0.05), rather than history (β = −0.04, *p* > 0.05), and state (β = −0.10, *p* > 0.05) of residential mobility, predicted a preference for cosmopolitan cities. Furthermore, the results of the mediation analysis showed that the indirect effect of relational mobility on the preference for cosmopolitan cities *via* dependence was statistically significant, as the bias-corrected bootstrap 95% CI excluded zero (β = 0.05; Boot SE = 0.02; 95% CI = [0.01, 0.10]). By contrast, the indirect effect of history (β = −0.01; Boot SE = 0.01; 95% CI = [−0.03, 0.10]), state (β = −0.01; Boot SE = 0.01; 95% CI = [−0.01, 0.03]), and intention (β = 0.01; Boot SE = 0.01; 95% CI = [−0.02, 0.02]) of residential mobility on the preference for cosmopolitan cities through dependence was not statistically significant, as the bias-corrected bootstrap 95% CI included zero.

## 4 Discussion

Based on a particular variant of the voluntary settlement hypothesis—the cosmopolitan settlement hypothesis—and the socio-ecological perspective in cultural psychology (Oishi et al., 2015; Sevincer et al., 2015, 2017; Oishi and Tsang, 2022), this study examined the relationships among relational mobility, personal residential mobility, independence, and Chinese senior undergraduates’ preference for cosmopolitan cities. The current study makes three contributions. First, to our knowledge, this is the first research to investigate the cosmopolitan settlement hypothesis in collectivistic Eastern cultures. Compared with prior studies (Sevincer et al., 2015, 2017) that examined adults or undergraduates from WEIRD societies, our study recruited Chinese senior undergraduates, who were choosing or would eventually choose their place of employment for the first time. Our research also systematically accounted for the effects of relational mobility and personal residential mobility. Thus, our study provides relatively robust evidence for the cosmopolitan settlement hypothesis in a collectivistic culture. Second, we also validated intention of residential mobility as a potential predictor of preference for cosmopolitan cities, which is unrecognized in previous work. Third, our research enhanced our knowledge of the mechanisms underlying relational mobility and Chinese senior undergraduates’ preference for cosmopolitan cities, with independent self-construal serving as the mediator. Overall, we extend previous psychological research on predictors of preference for cosmopolitan cities, from belief (e.g., Sevincer et al., 2015, 2017) and personality (Sevincer et al., 2017, 2021) to socioecological factors such as residential and relational mobility.

Consistent with Hypothesis 1, our results showed that dependence remained associated with a preference for cosmopolitan cities after controlling for demographic variables (i.e., gender, being an only child, subjective social status), the objective cosmopolitanism of one’s hometown and university cities, distance between the destination city and one’s hometown and university cities, as well as mobility (i.e., relational and residential mobility). On the one hand, this finding provides evidence for the cosmopolitan settlement hypothesis in a collectivist cultural context, which enriches the evidence for the hypothesis (Sevincer et al., 2015, 2017). On the other hand, our research controlled for other confounding variables that previous research on cosmopolitan settlement had not considered (Sevincer et al., 2015, 2017), providing more reliable evidence for the expectations. This suggests that the cosmopolitan settlement hypothesis may be universal and applicable not only to individualistic cultures but also to collectivistic cultures.

The cosmopolitan settlement hypothesis includes both self-selection and reinforcement mechanisms (Sevincer et al., 2015). Self-selection for settlement means that people with high independent self-construal are more inclined to self-select and voluntarily settle in cosmopolitan cities. Reinforcement refers to involvement in cosmopolitan settlement (e.g., adaptation and acculturation), which reinforces individuals’ orientation toward independence. Our findings indicated that Chinese senior undergraduates with high levels of independent orientation prefer to settle in cosmopolitan cities, supporting the self-selection mechanism. According to modernization theory, individualism will increase with the development of the economy and modernization (Greenfield, 2009). Indeed, research has shown that the cultural value of individualism is on the rise in contemporary China (Cai et al., 2019). Future studies could continue to investigate the self-selection and reinforcement mechanisms of the cosmopolitan settlement hypothesis.

Moreover, the direct effect of relational mobility on preference for cosmopolitan cities above and beyond related control variables was not solid, but the indirect effect of relational mobility on preference for cosmopolitan cities *via* independence was robust, partly supporting Hypothesis 2. The results revealed a mechanism—namely, dependence—that accounts for the within-culture influence of relational mobility on the preference for cosmopolitan cities. Relational mobility refers to one’s perception of the mobility of interpersonal and group relations in society. As relational mobility influences the degree to which people rely on others within solid or dynamic relationships, relational mobility may also impact the extent to which others are essential in an individual’s self-construal (San Martin et al., 2019). When placed into independent self-construal, individuals in high-mobility environments tend to define themselves as independent of others. The “city air” hypothesis posited that cities offer more freedom, fewer social restrictions, more opportunities, and higher relational mobility (Yamagishi et al., 2012; Yuki and Schug, 2020). Indeed, cosmopolitan cities embrace different ethnic and social groups, place more emphasis on equity and equality, and provide more opportunities to establish new interpersonal relationships and social support networks, symbolizing an independent lifestyle. In turn, independent individuals may be drawn to dwell in cosmopolitan cities due to the matching process between personality and location (Niedenthal et al., 1985).

Furthermore, although the indirect effect of residential mobility (i.e., history, state, and intention) on preference for cosmopolitan cities *via* dependence is close to zero and non-significant, the direct effect of intention of residential mobility on preference for cosmopolitan cities was small but robust, partly supporting Hypothesis 3. Previous studies showed that expectancy of future residential mobility rather than a history of residential mobility is linked to subjective wellbeing, insecurity, and materialism in selected Chinese undergraduates (Meng and Xie, 2019; Zhou and Xie, 2019). A recent review based mostly on research undertaken in Western cultures concluded that residential mobility not only makes individuals change their focus from communal to personal traits, but also alters their relationship preferences and values in favor of broader social relationships, more open interaction, and looser commitment groups (Oishi and Tsang, 2022). These contradictory findings imply that the effect of residential mobility may vary across cultures (Meng and Xie, 2019). China is a large agricultural country and one of the world’s founders of agricultural practices, with a low mobility rate. Most Chinese people are satisfied to live in their native land and are reluctant to move to another place. They also tend to hold that a person residing elsewhere will ultimately return to their native land. In recent years, however, due to the growth of the social economy, individuals are increasingly relocating to cities for further education and employment, and the mobility rate is rising. Because of past steady living conditions, Chinese people might not only see their lives as consistent, but may also not have a mobility mindset. This might explain why history and state of residential mobility was not linked to independence and preference for cosmopolitan cities. As research on residential mobility among the Chinese population is limited, more studies are warranted to understand the psychological and behavioral outcomes of residential mobility.

There are also some shortcomings in this study. First, the sample size of senior undergraduates from four universities selected in this study is small. Future research should increase the generalizability of the results by examining more varied and representative groups (e.g., employees). Second, this study measures cosmopolitanism of the preferred or intended city of employment rather than of the city that college graduates eventually choose after graduation. Although the preferred or intended city affects the actual city of employment, the city to which college students ultimately relocate is also influenced by factors such as cost of living, family characteristics, family influence, sustainable development, and employability (Sun et al., 2020; Zeng et al., 2021). Therefore, future researchers are encouraged to examine the applicability of the cosmopolitan settlement hypothesis in collectivist cultures after controlling for these factors. Third, since this research is cross-sectional, causal inferences cannot be drawn from the results. Future research can further explore the self-selection and reinforcement mechanisms of the cosmopolitan settlement hypothesis through experimental methods and longitudinal follow-up studies. Finally, individualism, for the Chinese undergraduates, may be different from that of students in Western cultures. Future studies should use qualitative data to reconceptualize individualism among Chinese young adults, and continue to investigate the connection between the preference for cosmopolitan cities and emerging forms of Chinese individualism.

## Data availability statement

The raw data supporting the conclusions of this article will be made available by the authors, without undue reservation.

## Ethics statement

The studies involving human participants were reviewed and approved by the Research Ethical Committee in Zhaoqing University. The patients/participants provided their written informed consent to participate in this study.

## Author contributions

HT: research design and protocol, raw manuscript, and manuscript revisions and corrections. GC: research protocol, data collection and analysis, and raw manuscript. ZL, RZ, and CL: data collection and manuscript revisions and corrections. YS: data analysis, raw manuscript, and manuscript revisions and corrections. All authors contributed to the article and approved the submitted version.

## Footnotes

1
https://www.geodatasource.com/demo
